# Single-Cell Profiling Identifies the SPDEF/GAS5 Axis as a Potential Driver of Tamoxifen Resistance in Estrogen Receptor-Positive Breast Cancer

**DOI:** 10.7759/cureus.111682

**Published:** 2026-06-28

**Authors:** Wen Jin, Claire Chen, Yanling Zhou, Jing Wang, Ren Hou, Wen-jie Shi

**Affiliations:** 1 Bioinformatics, Beijing SeekGene BioSciences, Beijing, CHN; 2 Medical School, Lawrenceville School, Lawrenceville, USA; 3 Eugenics and Genetics, Affiliated Hospital of Guilin Medical University, Guilin, CHN; 4 Medical Technology, Beijing Health Vocational College, Beijing, CHN

**Keywords:** breast cancer, gas5, single-cell rna sequencing, spdef, tamoxifen resistance

## Abstract

Background

Estrogen receptor-positive (ER+) breast cancer is the most prevalent breast cancer subtype, and tamoxifen remains the cornerstone of adjuvant endocrine therapy. However, a significant proportion of patients eventually develop acquired tamoxifen resistance, leading to disease recurrence and progression.

Methodology

To characterize the cellular and molecular mechanisms underlying this resistance at single-cell resolution, we performed an integrative analysis of publicly available single-cell RNA sequencing data from 16 ER+ breast cancer specimens, comprising 13 treatment-naive primary tumors and three tamoxifen-resistant recurrent tumors. Following rigorous quality control, batch correction, and dimensionality reduction, we identified 11 major cell populations in the tumor microenvironment. Copy number variation inference using InferCNV was utilized to confirm the malignant identity of the epithelial cells.

Results

Gene Ontology Biological Process enrichment analysis revealed that recurrent malignant cells were significantly enriched for pathways associated with translation, chromatin remodeling, and cell division compared with primary tumor cells. Sub-clustering of malignant epithelial cells resolved nine distinct subpopulations, among which a GAS5+ (growth arrest-specific 5-positive) subpopulation was markedly expanded in tamoxifen-resistant recurrent tumors. This subpopulation exhibited distinctive metabolic reprogramming characterized by enhanced aerobic respiration and energy metabolism. Monocle2-based pseudotemporal trajectory analysis positioned GAS5+ cells at a terminally differentiated state, likely derived from CLDN3+ and CEACAM6+ tumor progenitor cells. Single-cell regulatory network inference using SCENIC identified SPDEF (SAM pointed domain-containing ETS transcription factor) as a putative key transcription factor specifically active in GAS5+ tumor cells, and SPDEF expression was highly enriched in this subcluster. Validation in The Cancer Genome Atlas breast cancer cohort demonstrated that high SPDEF expression was significantly associated with worse overall survival (log-rank p = 0.011).

Conclusions

These findings implicate the SPDEF-GAS5+ regulatory axis as a potential driver of tamoxifen resistance and a compelling candidate for future therapeutic targeting in ER+ breast cancer.

## Introduction

Breast cancer is the most commonly diagnosed malignancy in women worldwide, with an estimated 2.3 million new cases and 685,000 deaths recorded in 2020 alone [1]. Estrogen receptor-positive (ER+) breast cancer, defined by the expression of estrogen receptor alpha (ERα, encoded by the *ESR1* gene), accounts for approximately 70-75% of all breast cancer cases and represents the largest molecular subtype [1,2]. Endocrine therapy targeting the estrogen signaling axis remains the cornerstone of treatment for ER+ disease. This includes selective estrogen receptor modulators such as tamoxifen, selective estrogen receptor degraders such as fulvestrant, and aromatase inhibitors [3,4]. Among these agents, tamoxifen has been utilized for decades; large randomized trials have demonstrated its efficacy in significantly reducing recurrence and mortality in both premenopausal and postmenopausal women with ER+ breast cancer [5].

Despite the established efficacy of tamoxifen, approximately 30-40% of patients eventually develop acquired resistance, leading to treatment failure and disease progression [3,6]. The mechanisms underlying tamoxifen resistance are multifactorial and include *ESR1* activating mutations that enable ligand-independent receptor activity, hyperactivation of downstream signaling pathways (e.g., PI3K/AKT/mTOR and MAPK/ERK), epigenetic reprogramming, and loss of ERα expression [6,7]. These diverse mechanisms collectively contribute to profound phenotypic plasticity and intratumoral heterogeneity, allowing resistant cell populations to evade the cytostatic effects of tamoxifen. However, the precise cellular subpopulations driving this resistance and the molecular regulators governing their fate remain incompletely understood.

Single-cell RNA sequencing (scRNA-seq) has emerged as a powerful technology to dissect the cellular complexity of tumors at an unprecedented resolution [8]. By profiling the transcriptomes of individual cells, scRNA-seq enables the deconvolution of tumor heterogeneity, the identification of rare cellular subpopulations, and the characterization of distinct functional states within the tumor microenvironment (TME) [9-11]. In breast cancer specifically, scRNA-seq has been successfully applied to map the immune landscape and identify tumor cell states associated with therapeutic responses [11,12].

GAS5 (growth arrest-specific 5) is a long non-coding RNA (lncRNA) initially identified as a growth arrest-induced transcript [13,14]. In the context of breast cancer, GAS5 generally functions as a tumor suppressor by promoting apoptosis and inhibiting cell proliferation. GAS5 expression is frequently downregulated in breast cancer compared to normal breast tissue, and its reduced levels have been linked to tamoxifen resistance [14,15]. Interestingly, GAS5 contains a structural motif that mimics the DNA-binding site for steroid hormone receptors, enabling it to act as a decoy to modulate glucocorticoid-induced gene expression relevant to tamoxifen response [14,15]. However, whether specific GAS5-expressing subpopulations emerge during acquired tamoxifen resistance, and how their transcriptional networks are rewired at the single-cell level, remains largely unexplored.

Transcription factors are master regulators of gene expression programs that dictate cell identity and functional states. The SAM pointed domain-containing ETS transcription factor (SPDEF), also known as PDEF, is an ETS family member preferentially expressed in epithelial tissues [16]. Initially characterized in the prostate epithelium as an activator of prostate-specific antigen (PSA) expression [16], SPDEF has subsequently been implicated in breast cancer, where its expression correlates with altered transcriptional programs and tumor progression [17]. Given that ETS transcription factors are among the most commonly deregulated transcription factor families in solid tumors [18], identifying their specific regulatory roles in resistant cellular niches is critical.

To elucidate the high-resolution cellular and molecular mechanisms underlying acquired tamoxifen resistance, we performed an integrative scRNA-seq analysis of treatment-naive primary and tamoxifen-resistant recurrent ER+ breast tumors. Specifically, our primary objectives were to: (1) identify and characterize distinct tamoxifen-resistant malignant epithelial subpopulations; (2) map the evolutionary trajectory of these cells using pseudotemporal analysis; and (3) discover potential master upstream transcription factors driving the resistance phenotype. Utilizing advanced computational approaches, we mapped the transcriptomic and metabolic landscape of resistant cells. Herein, we report the identification of a distinct, metabolically reprogrammed GAS5+ malignant subpopulation that is markedly expanded in tamoxifen-resistant tumors, and uncover SPDEF as its putative transcriptional regulator. Furthermore, our prognostic validation demonstrates that elevated SPDEF expression tightly correlates with poor clinical outcomes, underscoring its utility as a predictive biomarker for tamoxifen response. Our findings provide novel insights into the intratumoral heterogeneity of endocrine resistance and highlight the SPDEF/GAS5 axis as a compelling hypothesis for future therapeutic targeting in recurrent ER+ breast cancer.

## Materials and methods

Data acquisition

This study exclusively utilized previously published, de-identified, and publicly available data; therefore, institutional ethics committee approval was not required. Single-cell RNA sequencing (scRNA-seq) data comprising 16 ER+ breast cancer samples were obtained from the Gene Expression Omnibus (GEO) database (GSE240112, GSE245601). The dataset included 13 treatment-naive primary ER+ breast cancer samples (ER_Primary, denoted as PT1-PT13) and three tamoxifen-resistant recurrent ER+ breast cancer samples (ER_Recurrence, denoted as RT1-RT3), which represent unpaired independent samples from distinct patient cohorts. Additionally, bulk RNA-seq expression data and corresponding clinical information for breast cancer patients were acquired from The Cancer Genome Atlas Breast Invasive Carcinoma (TCGA-BRCA) cohort via the UCSC Xena browser.

Quality control and preprocessing

Preprocessing and quality control (QC) of the scRNA-seq data were performed using the Seurat R package (v4.3.0) [9]. For each sample, cells were retained for downstream analysis if they met the following stringent QC criteria: the number of uniquely expressed genes (nFeature_RNA) between 200 and 10,000, total unique molecular identifier (UMI) counts (nCount_RNA) greater than 500, and mitochondrial gene expression content below 20%. Following QC filtering, the gene expression matrices were log-normalized using the NormalizeData function with a default scale factor of 10,000. The top 2,000 highly variable features were then identified using the FindVariableFeatures function with the “vst” selection method. Subsequently, the data were centered and scaled using the ScaleData function.

Batch correction, dimensionality reduction, and cell clustering

To eliminate batch effects across different samples while preserving authentic biological variation, the Harmony algorithm was applied to the principal component analysis (PCA) embedding [19]. The first 30 Harmony-corrected dimensions were utilized for downstream clustering and dimensionality reduction. Uniform manifold approximation and projection (UMAP) was employed for two-dimensional visualization [20]. Unsupervised cell clustering was executed using the FindNeighbors and FindClusters functions in Seurat at a resolution of 1. Finally, cell types were manually annotated based on the expression of canonical marker genes: T cells (*CD3D*, *CD3E*), NKT cells (*NKG7*, *CD3D*), B cells (*MS4A1*), plasma cells (*JCHAIN*), endothelial cells (*VWF*, *PECAM1*), epithelial cells (*EPCAM*, *KRT19*), fibroblasts (*COL3A1*), macrophages (*C1QC*, *C1QA*), mast cells (*TPSAB1*), dendritic cells (*CD1E*), and mesenchymal stem cells (*ACTA2*, *RGS5*).

Copy number variation analysis using InferCNV

To distinguish malignant epithelial cells from non-malignant epithelial cells, InferCNV (Broad Institute, v1.0.4) was applied using T cells as the normal reference population [21]. Genes were arranged according to chromosomal position, and copy number variation (CNV) scores were inferred by computing the moving average of gene expression relative to the reference cells using a window size of 101 genes and a cutoff of 0.1. Inferred CNV scores were visualized as heatmaps, and their distribution was compared between epithelial cells and reference T cells to confirm the malignant identity of the epithelial population.

Identification of differentially expressed genes and functional enrichment analysis

To identify differentially expressed genes (DEGs) between malignant cells from the ER_Recurrence and ER_Primary groups, differential expression analysis was performed using the FindMarkers function in the Seurat package, employing the default Wilcoxon rank-sum test. Genes meeting the criteria of an adjusted p-value <0.05 and an absolute log2 fold change (|log2FC|) >0.25 were defined as significantly differentially expressed. Subsequently, Gene Ontology Biological Process (GOBP) enrichment analysis of these DEGs was conducted using the clusterProfiler R package (v3.18.1) [22]. To account for multiple comparisons, p-values were adjusted using the Benjamini-Hochberg method, and GOBP terms with a false discovery rate <0.05 were considered significantly enriched.

Tumor epithelial cell sub-clustering and subpopulation annotation

Malignant epithelial cells identified by InferCNV were extracted and re-clustered independently using Seurat. PCA was performed on the rescaled expression matrix, followed by UMAP visualization. Cell clustering was performed at a resolution of 0.8, yielding nine transcriptionally distinct malignant epithelial subpopulations. Each subcluster was annotated based on its most highly expressed marker genes determined by FindAllMarkers using the Wilcoxon test. The proportion of each subcluster in ER_Primary versus ER_Recurrence samples was calculated and visualized as a stacked bar chart.

Single-cell metabolic activity analysis using scMetabolism

Metabolic pathway activity at the single-cell level was quantified using the scMetabolism R package, which estimates pathway activity based on the expression of metabolic genes through an AUCell-based scoring algorithm [23]. A comprehensive panel of metabolic pathways curated from KEGG and REACTOME databases was assessed across all nine malignant epithelial subclusters. Metabolic activity scores were visualized as a bubble plot in which dot size represents the percentage of cells with detectable activity and color intensity represents the scaled mean activity score.

Pseudotemporal trajectory analysis using Monocle2

Pseudotemporal trajectory analysis was performed using Monocle2 (v2.26.0) [24,25]. To identify the robust gene sets driving the developmental progression, we applied a strict sequential filtering strategy. First, gene detection was determined using a minimum expression threshold (“min_expr = 0.1”). Next, we filtered the dataset to retain only those genes expressed in greater than 5% of the total malignant epithelial cells. Subsequently, differential gene expression analysis was performed on these retained genes across subclusters using the “differentialGeneTest” function. Genes demonstrating significant differential expression (qval < 0.01) were finally selected as the ordering genes. Malignant epithelial cells were then ordered along a pseudotime axis using the DDRTree dimensionality reduction method. Cell states were defined by the ordering function, and the root cell (pseudotime = 0) was set based on the expected differentiation trajectory. Hallmark gene set activity scores across cell states were calculated using the AUCell package with MSigDB Hallmark gene sets [26]. Gene expression dynamics along the pseudotime trajectory were examined for key genes including *SNHG12*. Branch expression analysis was used to identify genes differentially expressed between Cell Fate 1 and Cell Fate 2 branches relative to the pre-branch state.

Single-cell regulatory network inference using SCENIC

Transcription factor regulatory network analysis was performed using the pySCENIC pipeline (v0.12.1) [27]. The GRNBoost2 algorithm was used to infer co-expression modules from the single-cell expression matrix of malignant epithelial cells. Regulon candidates were filtered by transcription factor binding motif enrichment analysis using the cisTarget databases for human (hg19), and regulon activity was scored per cell using the AUCell method. Regulon Specificity Scores (RSS) were calculated for the GAS5+ malignant epithelial subpopulation to identify subcluster-specific transcription factors. Feature plots of SPDEF gene expression were generated in Seurat to visualize its spatial enrichment on the UMAP embedding.

The Cancer Genome Atlas survival analysis

Gene expression data and overall survival information for TCGA breast cancer (TCGA-BRCA) patients were obtained from the UCSC Xena browser. SPDEF expression levels (log2-transformed RSEM normalized counts) were used to stratify patients into SPDEF-high and SPDEF-low groups using the median expression value as the cutoff. Kaplan-Meier survival curves were generated for each group, and the log-rank test was used to compare survival distributions.

Statistical and bioinformatics analysis

A p-value below 0.05 was considered statistically significant. All basic statistical analyses were performed using R software (version 4.3.0). Furthermore, specific single-cell transcriptomic analyses and data visualizations were facilitated by the SeekSoul Online cloud analysis platform (SeekGene Biosciences, Beijing, China) [28].

## Results

Single-cell transcriptomic landscape of estrogen receptor-positive primary and tamoxifen-resistant recurrent breast cancer

**Figure 1:**
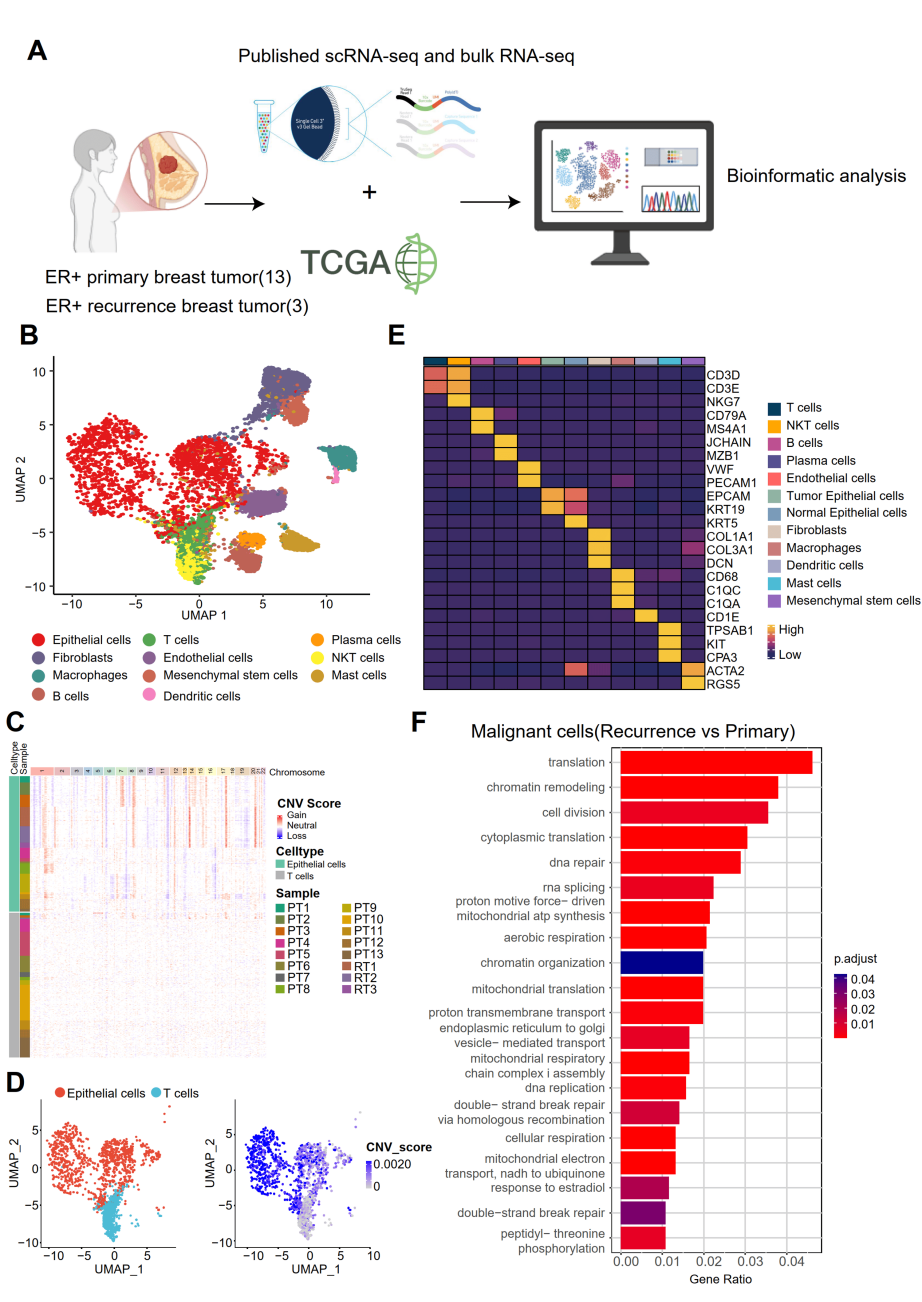
Single-cell transcriptomic landscape of ER+ primary and tamoxifen-resistant recurrent breast cancer. (A) Schematic overview of the study design. Publicly available scRNA-seq data from 16 estrogen receptor-positive (ER+) breast cancer samples (13 treatment-naive primary tumors (ER_Primary) and three tamoxifen-resistant recurrent tumors (ER_Recurrence)) were integrated with The Cancer Genome Atlas Breast Invasive Carcinoma (TCGA-BRCA) bulk RNA-seq data for comprehensive bioinformatics analysis. (B) Uniform manifold approximation and projection (UMAP) visualization of 11 major cell populations identified after integration, batch correction, and unsupervised clustering. (C) InferCNV heatmap displaying inferred copy number variation (CNV) profiles across chromosomal regions for malignant epithelial cells (top) and reference T cells (bottom) across primary tumors (PT1-PT13) and recurrent tumors (RT1-RT3). Gains are shown in red, losses in blue, and neutral regions in white. (D) UMAP plots showing the distribution of epithelial cells and T cells (left) and their inferred CNV scores (right), confirming the malignant identity of epithelial cells by higher CNV burden. (E) Dot plot showing expression of canonical marker genes for cell type annotation. Dot size indicates the percentage of expressing cells; color intensity indicates average expression level. (F) Bar chart showing Gene Ontology Biological Process (GOBP) enrichment analysis of differentially expressed genes in malignant cells from ER_Recurrence versus ER_Primary. Upregulated pathways are shown in red and downregulated in blue; color intensity represents the Benjamini-Hochberg-adjusted p-value.

**Figure 2:**
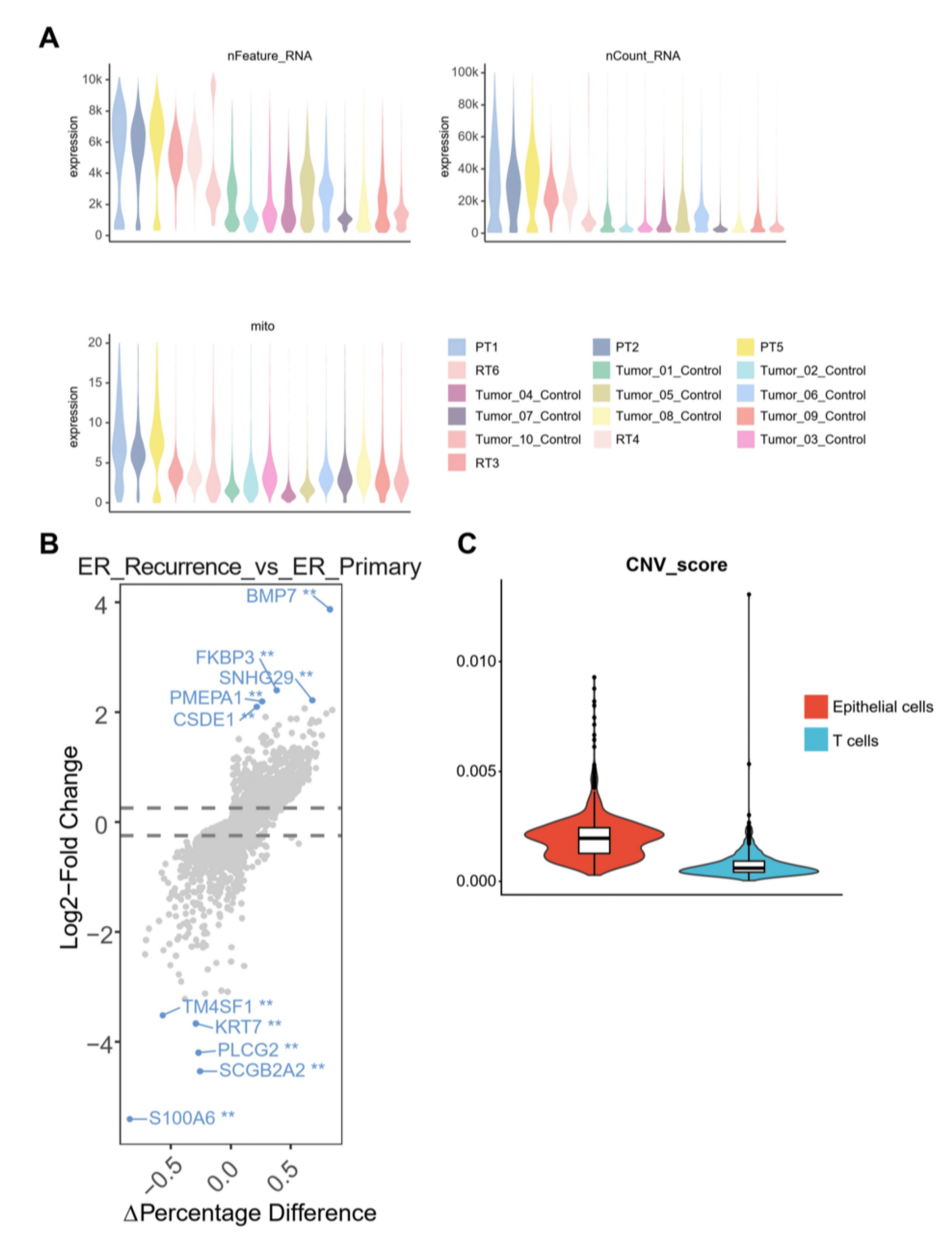
Quality control metrics, differential gene expression, and CNV score validation. (A) Violin plots displaying quality control metrics for all 16 estrogen receptor-positive (ER+) breast cancer samples after filtering: number of detected genes (nFeature_RNA), total unique molecular identifier (UMI) count (nCount_RNA), and percentage of mitochondrial gene expression (mito). (B) MA plot showing log2 fold-change versus percentage difference for genes differentially expressed in malignant cells from ER_Recurrence versus ER_Primary. Selected upregulated genes (*BMP7*, *FKBP3*, *SNHG29*, *PMEPA1*, *CSDE1*) and downregulated genes (*TM4SF1*, *KRT7*, *PLCG2*, *SCGB2A2*, *S100A6*) are labeled. (C) Violin plot comparing inferred copy number variation (CNV) scores between epithelial cells (red) and T cells (blue, reference), demonstrating higher CNV burden in epithelial cells consistent with their malignant identity.

To comprehensively characterize the cellular composition of ER+ breast cancer before and after tamoxifen-induced recurrence, we analyzed publicly available scRNA-seq data from a total of 16 samples: 13 treatment-naive primary ER+ breast tumors (ER_Primary) and three tamoxifen-resistant recurrent ER+ breast tumors (ER_Recurrence) (Figure 1, Panel A). Following stringent QC, including filtering based on the number of detected genes, total UMI counts, and mitochondrial gene fraction (Figure 2, Panel A), and Harmony-based batch correction, we performed unsupervised clustering and UMAP visualization of the integrated dataset.

This analysis revealed 11 distinct cell populations within the ER+ breast cancer TME (Figure 1, Panels B, E). Each population was annotated using canonical marker genes: epithelial cells (*EPCAM*+, *KRT19*+), T cells (*CD3D*+, *CD3E*+), NKT cells (*NKG7*+, *CD3D*+), B cells (*MS4A1*+), plasma cells (*JCHAIN*+), endothelial cells (*VWF*+, *PECAM1*+), macrophages (*C1QC*+, *C1QA*+), fibroblasts (*COL3A1*+), mast cells (*TPSAB1*+), dendritic cells (*CD1E*+), and mesenchymal stem cells (*ACTA2*+, *RGS5*+) (Figure 1, Panel E). The presence of these diverse stromal and immune populations is consistent with prior large-scale scRNA-seq studies in breast cancer that have documented a complex and heterogeneous TME [11,12]. The identification of these major cell types set the stage for focused analysis of the malignant epithelial compartment.

Extensive somatic copy number variations confirm the malignant identity of epithelial cells

Epithelial cells in scRNA-seq data represent a mixture of malignant tumor cells and normal epithelial cells, and their accurate distinction is critical for downstream analysis. We applied InferCNV to infer large-scale chromosomal CNVs from the scRNA-seq data, using T cells, which lack somatic copy number alterations, as the normal reference population [21]. The resulting CNV heatmap revealed extensive and heterogeneous large-scale chromosomal alterations across all individual tumor samples (Figure 1, Panel C), with distinct CNV patterns observed across the 13 treatment-naive primary tumors (designated as PT1 through PT13) and the three tamoxifen-resistant recurrent tumors (designated as RT1 through RT3). Epithelial cells exhibited significantly higher CNV scores compared to reference T cells (Figure 2, Panel C), confirming their malignant nature. UMAP visualization demonstrated that cells with elevated CNV scores clustered specifically within the epithelial cell cluster, whereas T cells showed near-zero CNV scores (Figure 1, Panel D). These findings validated the malignant identity of the epithelial cell population and justified their extraction for focused analysis.

Malignant cells in tamoxifen-resistant tumors exhibit enhanced translational activity and loss of estrogen responsiveness

To characterize the transcriptomic shifts driving the transition from primary to recurrent tumors, we compared the gene expression profiles of malignant cells between the ER_Recurrence and ER_Primary groups. Malignant cells from tamoxifen-resistant tumors exhibited a distinct transcriptional signature, characterized by the significant upregulation of genes such as *BMP7*, *FKBP3*, *SNHG29*, *PMEPA1*, and *CSDE1*, alongside the concomitant downregulation of *TM4SF1*, *KRT7*, *PLCG2*, *SCGB2A2*, and *S100A6* (Figure 2, Panel B). The marked induction of the lncRNA SNHG29 and the RNA-binding protein CSDE1 points to augmented post-transcriptional regulation in resistant cells. Conversely, the loss of classical luminal epithelial markers, including KRT7, SCGB2A2, and S100A6, strongly suggests a dedifferentiation program coupled with endocrine resistance.

Further functional enrichment analysis of these dysregulated genes illuminated the underlying biological adaptations. Malignant cells in the recurrent tumors fundamentally rewired their functional networks, showing a significant enrichment in pathways governing translation (adjusted p < 0.01), chromatin remodeling, cell division, DNA repair, and mRNA splicing (Figure 1, Panel F). Strikingly, genes downregulated in the recurrent state were predominantly associated with chromatin organization, mitochondrial translation, and the response to estradiol. This targeted suppression of estradiol responsiveness provides molecular confirmation of the loss of ER signaling dependence, a hallmark of tamoxifen resistance. Collectively, this profound transcriptomic reprogramming, encompassing hyperactive translational machinery, reinforced DNA repair, and epigenetic plasticity, reflects the evolutionary strategies by which these tumor cells evade the cytostatic pressure of tamoxifen and regain proliferative capacity [3,7].

A distinct GAS5+ malignant subpopulation is markedly enriched in tamoxifen-resistant tumors

**Figure 3:**
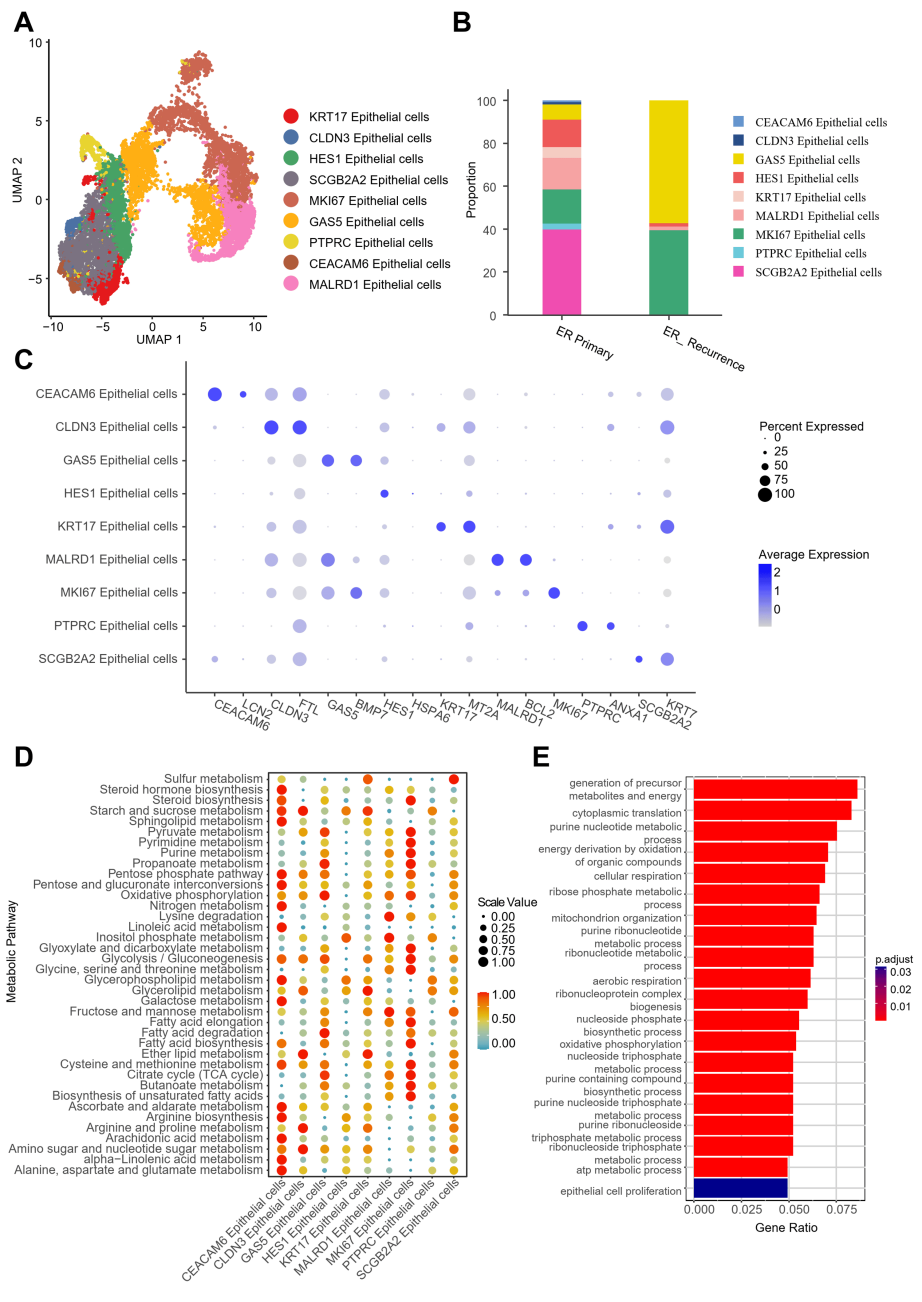
Characterization of malignant epithelial cell subclusters and identification of the GAS5+ subpopulation enriched in tamoxifen-resistant recurrent tumors. (A) Uniform manifold approximation and projection (UMAP) visualization of nine transcriptionally distinct malignant epithelial cell subclusters identified by sub-clustering analysis. (B) Stacked bar chart comparing the proportional composition of each malignant epithelial subcluster in ER_Primary (n = 13) and ER_Recurrence (n = 3) samples, highlighting the marked expansion of GAS5+ cells in the recurrence group. (C) Dot plot showing expression of signature marker genes for each malignant epithelial subcluster. (D) Bubble plot depicting metabolic pathway activity scores across nine malignant epithelial subclusters as quantified by the scMetabolism algorithm. (E) Bar chart showing Gene Ontology Biological Process (GOBP) enrichment analysis for GAS5+ epithelial cells compared to all other malignant epithelial subclusters.

Given the profound transcriptomic differences between primary and recurrent malignant cells, we next sought to characterize the intratumoral heterogeneity within the malignant epithelial compartment. High-resolution re-clustering of these cells unveiled nine transcriptionally distinct subpopulations, reflecting a highly complex and diverse tumor architecture (Figure 3, Panels A, C). These subclusters delineated a spectrum of functional states, ranging from actively proliferating tumor cells (such as the MKI67+ subpopulation) to more luminal-differentiated states characterized by classical epithelial markers (including the SCGB2A2+ and CLDN3+ subclusters). Notably, we also identified a unique subset of malignant cells defined by the high expression of GAS5 (the GAS5+ subpopulation). The coexistence of these diverse phenotypic states within the same TME highlights the pronounced plasticity of ER+ breast cancer, setting the stage for identifying specific cellular niches that may drive endocrine resistance.

To further investigate the dynamics of these malignant epithelial subclusters during disease progression, we compared their proportional compositions between the ER_Primary (n = 13) and ER_Recurrence (n = 3) cohorts. This analysis revealed a striking enrichment of the GAS5+ epithelial subpopulation in tamoxifen-resistant recurrent tumors (Figure 3, Panel B). Whereas GAS5+ cells constituted a relatively minor fraction of the primary tumor ecosystem, this subpopulation underwent a dramatic expansion in the recurrence group. In contrast, the proportions of more differentiated luminal-like cells, including the SCGB2A2+, KRT17+, and CLDN3+ subclusters, were markedly reduced in recurrent tumors.

The specific enrichment of the GAS5+ subcluster in tamoxifen-resistant disease strongly implicates this subpopulation in the development or maintenance of endocrine resistance. GAS5 is a well-characterized tumor suppressor lncRNA that typically promotes apoptosis and is known to be downregulated in bulk breast cancer tissues [13,14]. The paradoxical expansion of GAS5-expressing cells in the drug-resistant state suggests that this subpopulation may have evolved adaptive mechanisms to overcome GAS5-mediated growth suppression. Alternatively, it is plausible that GAS5 expression within these specific resistant niches serves a distinct, context-dependent functional role that diverges from its classical tumor-suppressive functions described in bulk tissue studies.

GAS5+ tumor cells undergo metabolic reprogramming characterized by enhanced oxidative phosphorylation

To characterize the functional properties of each malignant epithelial subcluster, we quantified single-cell metabolic pathway activity using the scMetabolism algorithm across all nine subclusters (Figure 3, Panel D). This analysis revealed broad metabolic heterogeneity among the malignant subclusters, with distinct pathway activity patterns delineating each subpopulation. Notably, GAS5+ cells exhibited particularly robust activity in pathways associated with oxidative phosphorylation, mitochondrial metabolism, and diverse biosynthetic processes.

This profound metabolic reprogramming, characterized by a shift toward heightened mitochondrial respiration, strongly implies that GAS5+ cells possess an enhanced capacity for energy production and macromolecular synthesis. Such metabolic flexibility is a well-established hallmark of cancer stemness and drug tolerance, enabling resistant tumor cells to survive the metabolic stress induced by endocrine therapy. The elevated oxidative phosphorylation dependency in this expanded GAS5+ subpopulation further corroborates its identity as a potential key driver of tamoxifen-resistant recurrence and highlights a metabolic vulnerability that could be therapeutically exploited.

To specifically define the transcriptional signature of GAS5+ cells relative to all other malignant subclusters, we performed GOBP enrichment analysis (Figure 3, Panel E). The top enriched pathways in GAS5+ cells included generation of precursor metabolites and energy, cytoplasmic translation, purine nucleotide metabolic process, energy derivation by oxidation of organic compounds, cellular respiration, ribose phosphate metabolic process, mitochondrial organization, purine ribonucleotide metabolic process, aerobic respiration, and ribonucleoprotein complex biogenesis (all p.adj < 0.05). Strikingly, epithelial cell proliferation was among the most significantly downregulated pathways in GAS5+ cells compared to other subclusters. This metabolic signature, characterized by enhanced aerobic respiration and oxidative phosphorylation coupled with reduced proliferation, suggests that GAS5+ tumor cells have undergone a metabolic shift reminiscent of a quiescence-like or drug-tolerant persister state. Such metabolic reprogramming toward enhanced mitochondrial activity has been observed in multiple cancer types as a mechanism of drug resistance [29,30].

GAS5+ tumor cells represent a terminally differentiated state associated with endocrine resistance

**Figure 4:**
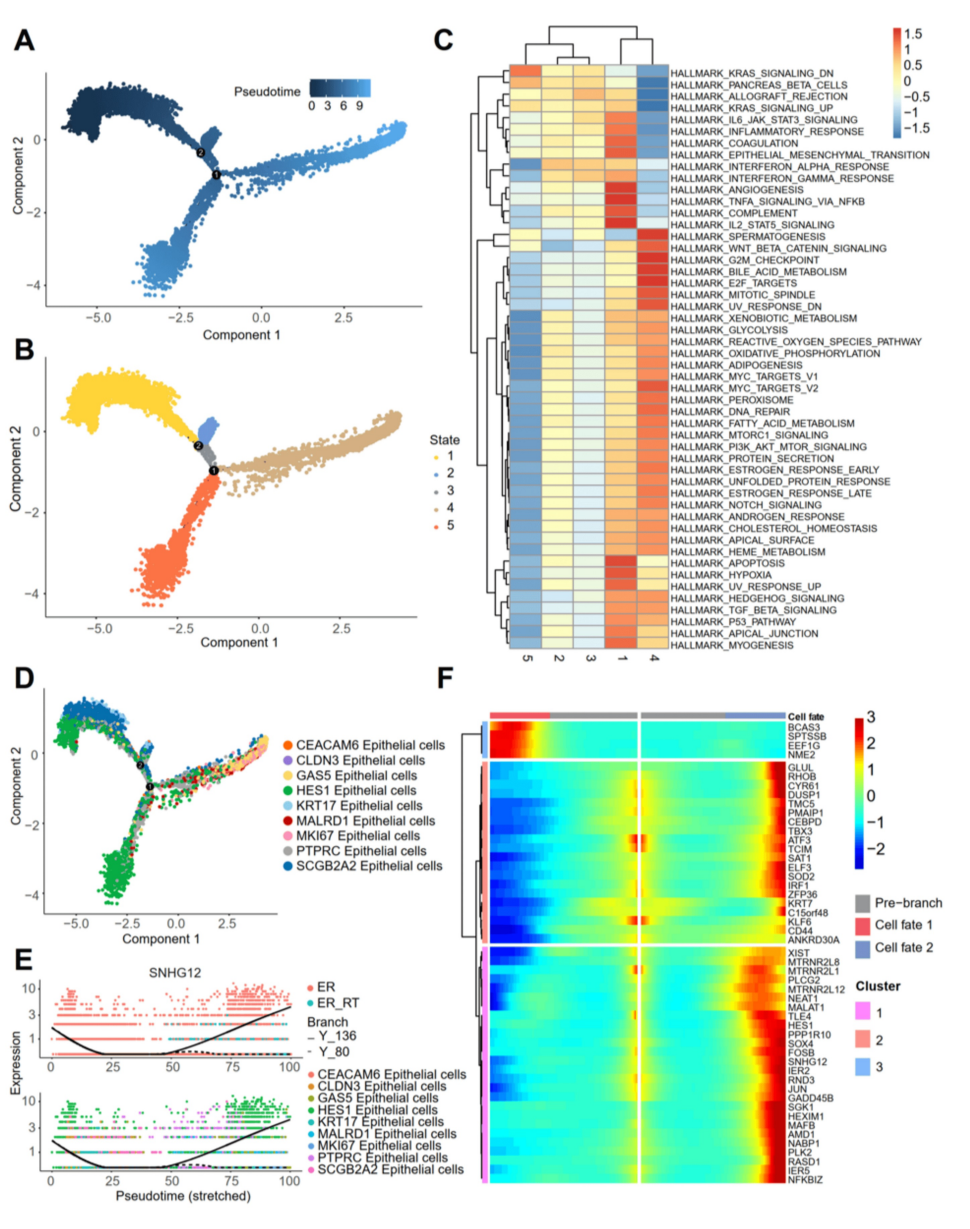
Pseudotemporal trajectory analysis reveals GAS5+ tumor cells as a terminally differentiated tamoxifen-resistant state. (A) Monocle2 pseudotime trajectory of malignant epithelial cells, colored by pseudotime value. (B) Monocle2 trajectory colored by cell state assignment (States 1-5). (C) Heatmap of Hallmark gene set activity scores across the five cell states. (D) Monocle2 trajectory plot colored by malignant epithelial subcluster identity, showing that GAS5+ cells occupy State 5 at late pseudotime. (E) Pseudotime-dependent expression dynamics of SNHG12 across ER and ER_RT sample groups and across epithelial subclusters along the two major trajectory branches (Y_136 and Y_80). (F) Heatmap depicting genes associated with the pre-branch state (gray), Cell Fate 1 (red), and Cell Fate 2 (pink). Genes are grouped into three clusters by hierarchical clustering.

The distinct transcriptomic and metabolic profile of GAS5+ cells raised the question of their position within the tumor cell differentiation hierarchy. To address this, we performed pseudotemporal trajectory analysis using Monocle2 [24], which orders cells along a developmental pseudotime axis based on transcriptional similarity (Figure 4, Panels A, B). The resulting trajectory defined five discrete cell states (States 1-5). Mapping of subcluster identity onto the DDRTree trajectory space (Figure 4, Panel D) revealed that GAS5+ cells were predominantly positioned in State 5, corresponding to late pseudotime values.

Heatmap analysis of Hallmark pathway activity across the five cell states (Figure 4, Panel C) revealed dynamic changes in key oncogenic and developmental signaling pathways along the trajectory, including KRAS signaling, IL6/JAK/STAT3 signaling, interferon responses, Myc targets, E2F targets, estrogen response, and transforming growth factor-beta signaling. Notably, estrogen response pathways progressively diminished from early to late pseudotime states, consistent with the progressive acquisition of endocrine resistance as cells differentiate toward the GAS5+ state. Analysis of pseudotime-dependent gene expression dynamics (Figure 4, Panel E) highlighted *SNHG12* as a key gene whose expression diverged between the two major trajectory branches (Y_136 and Y_80), with differential expression across ER and ER_RT groups and across malignant subclusters. Branch expression analysis (Figure 4, Panel F) identified three gene clusters characterizing the pre-branch state and the two cell fate destinations (Cell Fate 1 and Cell Fate 2), revealing distinct transcriptional programs governing divergent tumor cell fate decisions.

**Figure 5:**
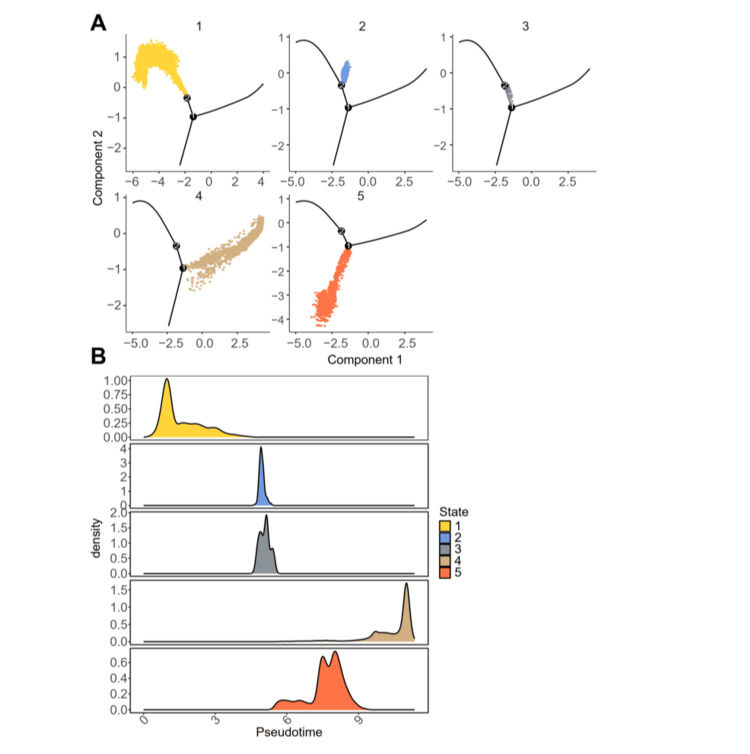
Pseudotime state distributions for malignant epithelial subclusters. (A) Individual Monocle2 pseudotime trajectory plots for each cell state (States 1-5), highlighting the distinct positioning of each state within the trajectory. (B) Density plots depicting the pseudotime value distribution of cells in each state (States 1-5), illustrating the temporal ordering of cell states along the trajectory. GAS5+ cells in State 5 exhibit a late pseudotime distribution.

To provide a more granular breakdown of the cellular distribution along the pseudotime axis, we examined individual pseudotime trajectory plots for each state (Figure 5, Panel A). State-specific pseudotime density distributions (Figure 5, Panel B) clearly demonstrated that States 1 and 4 (enriched for CEACAM6+ and CLDN3+ cells) occupy early pseudotime positions, while State 5 (enriched for GAS5+ cells) represents the terminal end of the trajectory. This spatial and temporal positioning strongly suggests that GAS5+ tumor cells represent a terminally differentiated state derived from more primitive tumor progenitor cells of the CLDN3+ or CEACAM6+ lineage under the selective pressure of endocrine therapy.

The transcription factor SPDEF promotes the transcriptional program of the GAS5+ subpopulation

The distinct gene expression program of GAS5+ cells implies the existence of upstream transcriptional regulators that maintain this cellular identity. To identify such master regulators, we applied the SCENIC pipeline for single-cell regulatory network inference [27]. SCENIC reconstructs gene regulatory networks by identifying co-expressed transcription factor-target gene modules and filtering them by transcription factor binding motif enrichment in gene promoter regions.

**Figure 6:**
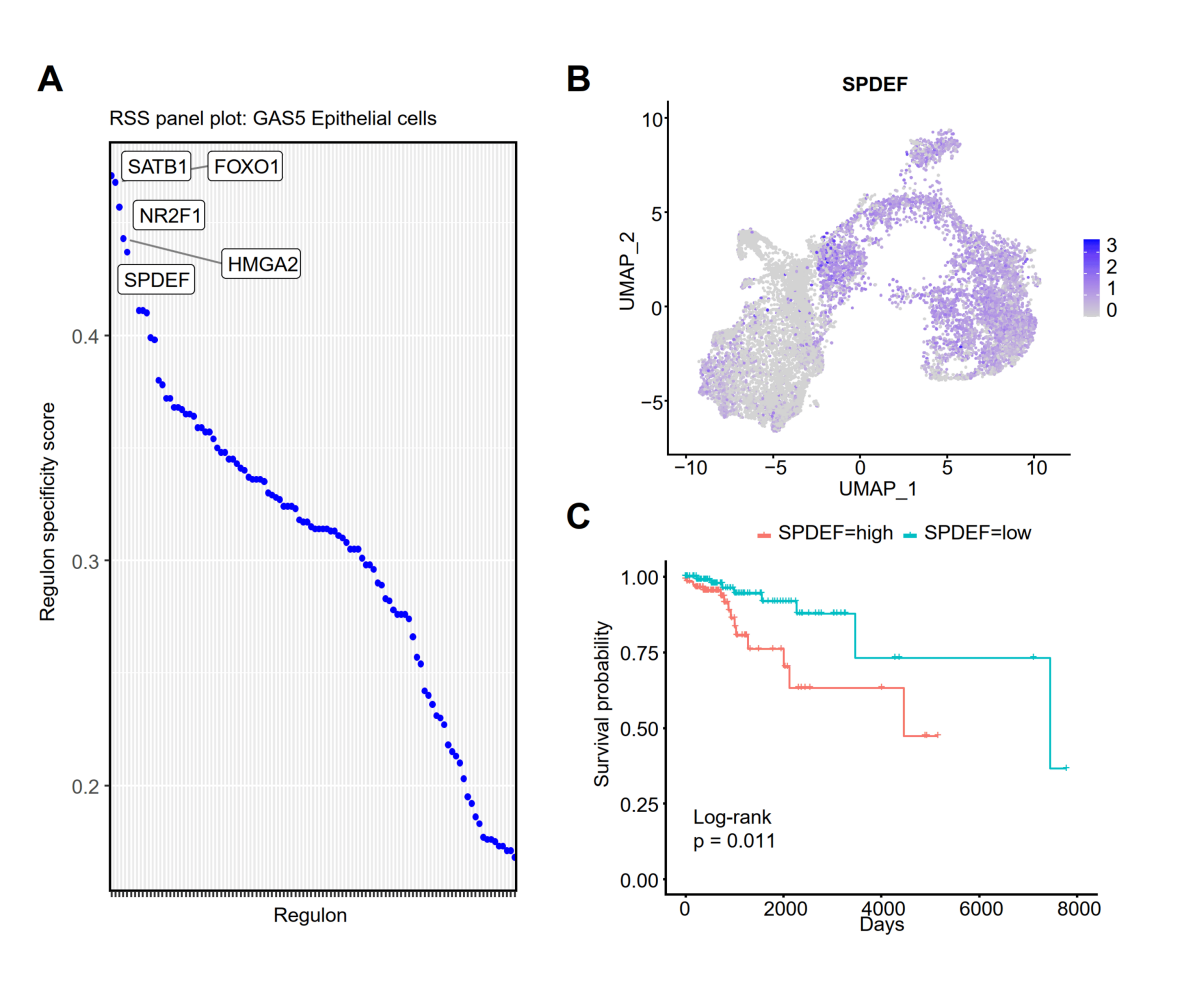
SCENIC identifies SPDEF as a putative master regulator of GAS5+ tumor cells, with high expression predicting poor prognosis in TCGA breast cancer. (A) Regulon Specificity Score (RSS) panel plot for GAS5+ epithelial cells from the SCENIC analysis. Regulons are ranked by their specificity scores for the GAS5+ subcluster, with top regulons labeled (*SATB1*, *FOXO1*, *NR2F1*, *HMGA2*, *SPDEF*). (B) Uniform manifold approximation and projection (UMAP) feature plot showing SPDEF gene expression level across all malignant epithelial cells, demonstrating enrichment within the GAS5+ subcluster. Color intensity indicates expression level (light: low; dark purple: high). (C) Kaplan-Meier overall survival curves for The Cancer Genome Atlas Breast Invasive Carcinoma (TCGA-BRCA) patients stratified by SPDEF expression level (SPDEF-high, red vs. SPDEF-low, teal). Statistical significance was assessed by the log-rank test (p = 0.011).

The RSS plot for GAS5+ epithelial cells (Figure 6, Panel A) ranked all inferred regulons by their specificity to this subcluster. The top subcluster-specific regulons included *SATB1*, *FOXO1*, *NR2F1*, *HMGA2*, and *SPDEF*. Among these, SPDEF emerged as a particularly compelling candidate: it is an ETS family transcription factor known to regulate gene expression in epithelial cells [16,17], and the ETS family represents one of the most frequently deregulated transcription factor families in human cancer [18]. Feature plot visualization of SPDEF expression across all malignant epithelial cells confirmed that SPDEF transcripts were highly enriched specifically within the GAS5+ subcluster (Figure 6, Panel B), with high expression concentrated in the GAS5+ cell region of the UMAP embedding while other subclusters showed substantially lower expression. The spatial concordance between SPDEF expression and GAS5+ subcluster identity, together with its high RSS specificity score, identifies SPDEF as a putative master transcriptional regulator of this tamoxifen-resistant subpopulation.

Elevated SPDEF expression predicts poor overall survival in breast cancer patients

To determine the clinical significance of SPDEF overexpression, we analyzed transcriptomic and survival data from the TCGA-BRCA cohort. Patients were stratified into SPDEF-high and SPDEF-low groups based on median expression levels. Kaplan-Meier survival analysis demonstrated that patients in the SPDEF-high group experienced significantly shorter overall survival compared to those in the SPDEF-low group (log-rank p = 0.011) (Figure 6, Panel C).

These data provide compelling clinical evidence that elevated SPDEF expression serves as an adverse prognostic factor in breast cancer, corroborating its potential oncogenic role in driving tamoxifen-resistant tumor cell subpopulations. The convergence of our single-cell regulatory network findings, which pinpoint SPDEF as a specific regulator of GAS5+ resistant cells, with bulk tumor survival data strongly positions the SPDEF-GAS5+ axis as a clinically relevant candidate for therapeutic targeting in ER+ breast cancer.

## Discussion

In the present study, we performed a comprehensive scRNA-seq analysis of 16 ER+ breast cancer specimens to delineate the cellular and molecular mechanisms underlying tamoxifen resistance. Our transcriptomic profiling revealed that recurrent tamoxifen-resistant malignant cells undergo profound reprogramming, characterized by enhanced translation, chromatin remodeling, and cell division, alongside a concomitant loss of estrogen responsiveness. Within these recurrent tumors, we identified a marked enrichment of a distinct GAS5+ malignant epithelial subpopulation exhibiting a unique metabolic phenotype driven by elevated aerobic respiration and energy metabolism. Pseudotemporal trajectory analysis further demonstrated that these GAS5+ cells represent a terminally differentiated tumor state, which appears to evolve from more primitive CLDN3+/CEACAM6+ progenitor cells under the selective pressure of endocrine therapy. To uncover the regulatory mechanisms maintaining this resistant state, SCENIC network inference identified SPDEF as a putative master transcription factor governing the GAS5+ cellular identity. The clinical relevance of this regulatory axis was ultimately corroborated by TCGA cohort data, demonstrating that high SPDEF expression is significantly associated with worse overall survival in breast cancer patients.

Our identification of a GAS5+ tumor cell subpopulation markedly enriched in tamoxifen-resistant recurrent ER+ breast cancer is particularly intriguing in light of the established literature on GAS5 function. GAS5 is a well-characterized tumor suppressor lncRNA that promotes apoptosis and inhibits cell proliferation, and its expression is downregulated in breast cancer tissues compared to normal mammary gland [13,14]. Moreover, GAS5 contains sequences that mimic steroid hormone response elements, thereby acting as a decoy for glucocorticoid receptor binding and modulating glucocorticoid-mediated gene expression relevant to tamoxifen response [14,15]. Paradoxically, our single-cell analysis reveals that GAS5 is used as a marker gene for a subpopulation that expands under tamoxifen-resistant conditions. This apparent contradiction may be explained by cell-type-specific differences in GAS5 function: the GAS5+ subpopulation may have evolved mechanisms that neutralize GAS5-mediated tumor suppression while exploiting GAS5-driven transcriptional programs for survival under endocrine pressure. Alternatively, the high GAS5 expression in this subcluster may reflect a unique cellular state not captured in bulk tissue studies, underscoring the importance of single-cell resolution for understanding lncRNA biology in cancer.

The GOBP enrichment profile of GAS5+ cells, characterized by upregulation of aerobic respiration, oxidative phosphorylation, and mitochondrial organization, is consistent with a drug-tolerant persister (DTP) cell phenotype. DTPs have been described across multiple cancer types as slowly proliferating, metabolically active cells that survive drug exposure through enhanced mitochondrial activity and epigenetic mechanisms [7,30]. The observed reduction in epithelial cell proliferation pathways in GAS5+ cells further supports their quiescence-like character. This metabolic signature distinguishes GAS5+ cells from the actively proliferating MKI67+ subpopulation and may underlie their selective advantage under tamoxifen treatment, as tamoxifen preferentially targets actively dividing cells. The aerobic respiration enrichment also echoes the Warburg effect reversal observed in some resistant cancer cells, which shift from glycolysis back toward oxidative phosphorylation as a survival mechanism [30].

The identification of SPDEF as a putative master regulator of GAS5+ tumor cells by SCENIC analysis is a central finding of this study. SPDEF, also known as PDEF, belongs to the ETS family of transcription factors, which are among the most frequently deregulated transcription factor families in human cancer [18]. SPDEF was initially characterized as a prostate epithelium-specific ETS transcription factor that transactivates PSA gene expression [16]. Subsequent investigation in breast cancer by Feldman et al. demonstrated that PDEF/SPDEF expression correlates with invasive potential and is associated with altered transcriptional programs including upregulation of genes implicated in cancer progression [17]. In epithelial cancers, SPDEF regulates a transcriptional network that includes genes involved in cell differentiation, motility, and metabolic reprogramming, suggesting pleiotropic oncogenic functions in diverse cancer types. Our TCGA validation data, demonstrating that SPDEF-high expression predicts significantly worse overall survival in breast cancer patients (p = 0.011), provide independent clinical corroboration that SPDEF is associated with adverse outcomes in this disease. Collectively, these findings suggest the hypothesis that SPDEF functions as a potential oncogenic transcription factor in ER+ breast cancer by driving a tamoxifen-resistant GAS5+ cell state, likely through coordination of the metabolic and transcriptional reprogramming we observed in this subpopulation.

The pseudotime trajectory analysis provides additional mechanistic insight by revealing the differentiation hierarchy of malignant epithelial subclusters. The positioning of GAS5+ cells at the terminal end of the pseudotime trajectory, arising from early-stage CLDN3+ and CEACAM6+ tumor progenitor states, suggests a model in which tamoxifen treatment creates selective pressure that drives tumor cell differentiation along a specific trajectory toward the GAS5+/SPDEF-high resistant state. The progressive loss of estrogen response pathway activity along the pseudotime axis is consistent with the gradual acquisition of endocrine resistance during this differentiation process. *SNHG12* emerged as an important pseudotime-dependent gene, consistent with its established roles in promoting cancer cell survival across multiple tumor types. These findings collectively support a computational model in which the SPDEF-GAS5+ axis represents a terminal differentiation program adopted by tamoxifen-resistant ER+ breast cancer cells.

Several limitations warrant acknowledgment. First, the number of tamoxifen-resistant recurrent samples (n = 3) is relatively small, and larger cohorts with longitudinal sampling before and after tamoxifen treatment would strengthen conclusions about the dynamics of GAS5+ cell expansion during resistance development. Second, as this study relied exclusively on computational inference from publicly available data, the proposed SPDEF-GAS5 regulatory axis remains a strong hypothesis rather than a definitive causal mechanism. Direct experimental validation, including loss-of-function studies, ChIP-seq to map SPDEF target genes, and in vivo tamoxifen resistance models, will be essential to establish biological causality before this axis can be considered a definitive therapeutic target. Third, spatial transcriptomic data would be valuable to characterize the microenvironmental niche of GAS5+ cells and their interactions with stromal and immune populations. Fourth, the functional consequences of GAS5 expression in this specific subpopulation relative to its established tumor suppressor role in bulk cancer remain to be clarified by future mechanistic studies.

## Conclusions

This study provides a single-cell resolution map of the transcriptomic landscape of tamoxifen-resistant ER+ breast cancer and characterizes a GAS5+ malignant epithelial subpopulation, enriched in recurrent tumors, endowed with a distinctive oxidative metabolic phenotype, and potentially governed by the transcription factor SPDEF, as a putative cellular mediator of endocrine resistance. The adverse prognostic significance of SPDEF in the TCGA breast cancer cohort highlights its potential as a compelling candidate for therapeutic targeting and a biomarker of tamoxifen resistance. Future experimental studies aimed at functionally validating and pharmacologically targeting SPDEF activity, or exploiting the metabolic vulnerabilities of GAS5+ cells, may offer new strategies to overcome endocrine resistance and improve outcomes for patients with ER+ breast cancer.
